# Pig-Islet Xenotransplantation: Recent Progress and Current Perspectives

**DOI:** 10.3389/fsurg.2014.00007

**Published:** 2014-03-24

**Authors:** Hai-Tao Zhu, Wan-Li Wang, Liang Yu, Bo Wang

**Affiliations:** ^1^Department of Hepatobiliary Surgery, First Affiliated Hospital, Medical College, Xi’an Jiaotong University, Xi’an, China

**Keywords:** islet, pig, isolation, xenotransplantation, immune, rejection

## Abstract

Islet xenotransplantation is one prospective treatment to bridge the gap between available human cells and needs of patients with diabetes. Pig represents an ideal candidate for obtaining such available cells. However, potential clinical application of pig islet still faces obstacles including inadequate yield of high-quality functional islets and xenorejection of the transplants. Adequate amounts of available islets can be obtained by selection of a suitable pathogen-free source herd and the development of isolation and purification method. Several studies demonstrated the feasibility of successful preclinical pig-islet xenotransplantation and provided insights and possible mechanisms of xenogeneic immune recognition and rejection. Particularly promising is the achievement of long-term insulin independence in diabetic models by means of distinct islet products and novel immunotherapeutic strategies. Nonetheless, further efforts are needed to obtain much more safety and efficacy data to translate these findings into clinic.

## Introduction

Diabetes is one of the most dangerous threats to human health. However, pancreatic islet transplantation has gradually showed satisfactory and prospective application in the treatment of type 1 diabetes mellitus (T1DM) (1). In the year 2000, Edmonton protocol (2) demonstrated that islet allotransplantation had achieved a remarkable success, but shortage of donors still prevented the progression of clinical islet transplantation. Xenotransplantation provides an effective and appropriate solution for this limitation. Among the potential candidates for islet xenotransplantation, pig is considered as the most ideal donor for future clinical applications (3–8). Although encouraging findings have been obtained in pig-to-primate islet xenotransplantation (9–11), the potential clinical application of pig islet still faces two major challenges: inadequate supply of islet cells with high-quality and xenorejection. This review will discuss the current approach and progress in pig donor selecting, isolation and preparation of pig-islet grafts, prevention of xenorejection, microbial safety, and obtained findings of clinical trials.

## Original of Porcine Islets

Islets obtained from embryonic, fetal, neonatal, young, or adult pigs have been selected as the grafts for xenotransplantation. Despite several years of study, no exact consensus has been achieved about the selection of the most optimal pig to supply adequate viable isolated islet cells for preclinical xenotransplantation (11, 12). Only islet xenografts harvested from neonatal (2–3 days old) and adult (>6 months) pigs have been shown to correct diabetes in non-human primates (NHPs) or humans (13–16).

Fetal pig islet-like cell clusters (ICCs) and neonatal pig islets (NPIs) are immature cells, which can be easily obtained by enzymatic digestion and simple culture. Other advantages of ICCs and NPIs are their apparent resistance to ischemic and inflammatory damage during isolation that makes islet recovery more efficient. However, several studies suggested that ICCs had poor insulin response to glucose (17–21), Typically, ICCs requires 2–3 months for maturation to achieve *in vivo* functionality (22). Additionally, in diabetic monkeys, transplanted pig ICCs were almost completely destroyed within 12 days post-transplantation (23). All the disadvantages restrict the potential clinical application of pig ICCs.

Neonatal pig islets consist of differentiated pancreatic endocrine cells (about 35%) and primarily epithelial cells (about 57%), which is also considered as islet precursor cells (24, 25). NPIs are more responsive to high glucose than ICCs and subsequently have a powerful functional ability to restore normoglycemia in diabetic animals, which are mainly due to β cell expansion and the striking differentiation of epithelial cells into β cell (26–29). NPIs clearly express xenoantigens including sialic acid antigens, Hanganutziu–Deicher (H–D) antigens, and Galα1–3Galβ1–4GlcNAc-R (α-Gal) epitopes (30). However, with the development of genetic engineering technology, stable gene transferred NPIs can therefore effectively attenuate the xenoantigenicity (30–32). In pig-islet xenotransplantation, several studies suggested that cell numbers in the range of 25,000–100,000 islet equivalents (IEQ)/kg recipient body weight were required to achieve insulin independence in diabetic NHPs (13, 16, 33–35). Usually, after *in vitro* culture, tissue from one neonate pancreas yields about 50,000 NPI aggregates (24); thus, at least four neonate pig donors are required to treat a diabetic primate weighing 6–8 kg.

Adult pig is regarded as the major donor source of islet xenografts, which can supply a sufficient number of viable islet cells and start functioning immediately after transplantation. More than 255,000 adult pig islets (APIs) with high purity (80–95%) can be isolated from an adult pig donor (36). Furthermore, published study also reported an extremely high APIs yield, up to 800,000 IEQ per pancreas after purification (37). The achievements make it possible to perform single pig donor clinical xenogeneic transplantation. Additionally, in comparison with young pig (<6 months) pancreas, a great number of large (150–200 μm) and well-structured islets can be obtained from adult pig donors (38–40). In large islets, the centralized structure for both collagen and capillaries could reduce enzymatic digestion-induced islet damage and subsequently facilitate post-transplant revascularization (38, 41). Consequently, APIs possess a better potential for cellular engraftment in xenotransplantation.

The breed and strain of donor pigs have a vital impact on the outcome of islet isolation. Previous studies suggested that German Landrace and Large White pigs appeared to be more suitable islet donor breeds than Duroc, Pietrain, Hampshire, Belgium Landrace, local farmers (hybrid), and wild-type pigs (42, 43). The German Landraces showed the highest numbers of large islets (150 μm) and islet volume density (%) (43). In contrast, Heiser et al. reported that Pietrain pig could produce more islet yields than purebred German Landrace, Munich minipig “Troll,” and hybrid pigs (44). The variability of results in different laboratories was possibly related to alterations in islet isolation and preparation procedure. Recently, very high islet yields (up to 9,589 ± 2,838 IEQ/g pancreas) with large size and well-function were harvested from adult Chicago Medical School (CMS) miniature pigs (45). The CMS miniature pigs can be bred under specific pathogen-free (SPF) conditions. All these making this pig breed potentially a better donor candidate for future clinical islet xenotransplantation.

## Isolation and Preparation of Porcine Islets

Islet-like cell clusters and NPIs can be easily obtained by simple enzymatic digestion and subsequent pre-transplantation culture due to relative lack of exocrine tissues and concomitant relative abundance of endocrine tissues (23, 24, 46). Briefly, the pancreas from fetuses or neonates is surgically removed in sanitary environments, chopped into small fragment measuring 1–2 mm^3^, digested by collagenase, washed, and then explanted in Petri plate for culturing. Normally, a culture time of 4–9 days is required to clear exocrine cells and facilitate islet cell re-aggregation. The isolation and preparation of APIs grafts from adult pigs is similar to that of humans. Factors including quality status of donor pancreas, blood exsanguinations, warm ischemia time (WIT), perfusate, types of digestive enzyme, and isolation/purification process will affect the islet yield and function (47–49).

### Selection and procurement of pancreas

Morphological screening before isolation process is necessary to obtain amounts of islet cells with high-quality, decrease variance in islet yield and viability, and reduce economic costs. A rapid and inexpensive strategy for assessment of pig donor pancreas was established in 1994 (50), which indicated that a pancreas containing round or oval islets with compact borders would provide successful islet isolation. Meanwhile, islet size *in situ* was also regarded as another important parameter for successful isolation. A donor pancreas with predominantly large islets (>200 μm) generally yielded significantly higher numbers of cell grafts (51, 52). Additionally, a recent study indicated that only islet equivalents per mm^2^ (IE/mm^2^) in splenic lobe of pancreas could dramatically predict an accurate islet yield, while variables such as pig donor age, gender, ischemic time, and enzyme lot were not significantly correlated with islet yield (53).

Warm ischemia time during pancreas procurement should be reduced as much as possible to prevent autolysis of pig donor pancreas and apoptosis in islet cell, reduce expression of inflammatory mediators, and improve islet survival rate during culture (54, 55). However, there is still lack of uniform standards of safe WIT for pig-islet preparation. It is considered that WIT within 10 min was essential for successful pig-to-primate islet xenotransplantation (48).

### Isolation of porcine islets

Although several major improvements or modifications have been made in the field of pig pancreas digestion and islet isolation (56–58), there is still a need for better isolation methods. Usually, immediately after harvesting of intact pig pancreas under sterile conditions, the pancreatic duct is cannulated and then collagenase is delivered by syringe or controlled perfusion after cold preservation (<2 h of cold ischemia time is advisable). Currently, a novel good manufacturing practices (GMP) grade bovine nervous tissue-free enzyme, Liberase MTF C/T, which contains lower endotoxin content (<10 EU/mg), is recommended for successful pancreas digestion (59, 60). Following the step of collagenase injection, the pancreas is placed in a new re-circulating digestion/filtration chamber (called Oxford chamber), which is similar to standard Ricordi chamber. The Oxford chamber results in less destruction of tissue, greater yield of islets, as well as improved cell viability (61). With the new device, up to 5,000 islets/g pancreas can be obtained from juvenile pigs. During this digestion process, another study recommended that digestion time should be limited to 35 min and temperature in the chamber should not exceed 35°C (62). The limited time and lower temperature avoid the deleterious impact of overdigestion and oxidative stress induced islet damage, respectively (45, 63). Once the islets are dissolved from collagen matrix, the freshly isolated cells are immediately removed from digest/filtration chamber and then placed in a cell processor (COBE 2991) for purification.

### Purification of porcine islets

Purification is the next necessary process to completely separate islets from acinar tissues, especially for islet preparation from young or adult pigs. The classical purification method is based on a density gradient centrifugation, taking advantage of the fact that the density of islet is lower than that of exocrine tissue. The final purity of islet products mainly depends on the characteristics of density gradients (64). At present, Ficoll is the most commonly used reagent for islet purification (56, 65), and usually a purity of 70–90% (islets/whole pancreas) can be achieved (66). However, this reagent has disadvantages of hypertonicity, high viscosity, and possible endotoxin content, which are harmful to pig-islet viability and function (67). In contrast, Iodixanol is widely used in clinical examination as an iso-osmotic contrast medium (approximately 290 mOsm/kg), which is free of endotoxin. Compared with Ficoll solution, Iodixanol can significantly improve pig-islet yield and viability, reduce cytokine/chemokine generation, and prevent islet mass loss during pre-transplantation culture. (67–69).

In general, freshly obtained islets from adult pigs are often of heterogeneous constitution, culturing provides a valuable tool to improve xenograft quality and homogeneity (70). Although islet recovery decreased dramatically after prolonged culture (7–14 days), the APIs displayed shorter time-to-normoglycemia and reversed hyperglycemia in all recipients.

## Immunological Rejection of Porcine Islet Xenotransplantation

### Instant blood-mediated inflammatory reaction

Immunological rejection, which poses negative impacts on islet engraftment as well as function, is still a major obstacle for successful clinical application of pig-islet xenotransplantation (71, 72). Several studies showed that after intraportal injection, tissue factor (TF) produced and expressed on the transplanted pig islets would first trigger platelet accumulation, coagulation, and complement activation, neutrophil infiltration, as well as graft dysfunction and destruction when exposed to fresh recipients’ blood; this phenomenon was described as instant blood-mediated inflammatory reaction (IBMIR) (73–75). Generally, IBMIR contributes to a considerable early pig-islet xenograft loss (estimated up to 60–80%) in diabetic primate (71, 76). Thus, effective treatments targeting IBMIR response provide promise for minimizing the critical islet dose to restore normoglycemia and insulin independence. After IBMIR has emerged, other subsequent immune responses intervene more specifically in relation to pig-islet xenografts.

### Hyperacute rejection

Islet engraftment is a process of graft revascularization mainly by recipients’ endothelial cells, very few endothelial cells from donors can survive after pre-transplantation culture (77). In addition, Gal molecules expressed on pig islets are lower than solid-organs, only 5% of Gal is expressed on the surface of APIs and α1,3-galactosyltransferase (α1,3GT) activity was also undetectable (78, 79). Hence, the pig-islet xenografts rarely undergo hyperacute rejection (HAR) as observed in vascularized organ transplants. Furthermore, in a study of pig-to-NPH islet xenotransplantation, neither increase in Gal-specific IgG or IgM antibody levels nor Gal-specific staining (isolectin B4) on islets was observed (16). All the data indicate that natural anti-Gal antibodies do not appear to play a major role in the immune rejection of APIs in diabetic NHPs. Nevertheless, Gal expression on pig islets is age dependent, both ICCs and NPIs clearly express a relatively higher level of Gal antigens (up to 11–19% of total islets) (30, 78). Additionally, the Gal expression remains positive with both small (<100 μm) and large islets (>100 μm) after isolation procedure (41). Therefore, Gal molecules are still considerable targets for humoral xenorejection.

### Cellular rejection

Still, if the islet xenografts escape the acute damages due to IBMIR and additional humoral response, they will be subject to acute cellular rejection. Typically, in pig-to-rodent islet xenotransplantation, cellular rejection appears to be mainly a CD4^+^ T-cell-dependent process (80–82). In diabetic primates, the acute cellular rejection takes place during the first 24 h to 20 days after transplantation and is characterized by a massive infiltration of macrophages and T cells (CD4^+^ and CD8^+^ T cells) in the periphery of grafts (16, 83). Lindeborg et al. further demonstrated that the CD4^+^ T cells were the major phenotype of activated T-cell clones reactive against pig-islet antigens (84). Besides, the T-cell-mediated response possibly induces numerous other cellular responses such as natural killer cell (NK cell), B cell, and innate responses. All these indicate that T cell plays a crucial and central role in the cellular rejection against pig islets. Although pig-islet cells are not believed to act as professional antigen presenting cells (APCs), both direct and indirect pathways of antigen presentation appear to be involved in the xenogeneic T-cell response (72). Usually, T cells require two signals to become fully activated, one is T-cell receptor (TCR) signaling, and the other is co-stimulatory signal. Co-stimulation signal, which is provided by interaction between co-stimulatory molecules expressed on the membrane of APC and T cell, is very crucial to induction and amplification of an effective immune response (85). Thus, therapies targeting different pathways affecting T-cell activation are believed to induce a long-term pig-islet survival and host hyporeactivity.

## Methods to Relieve Xenogeneic Rejection

### Encapsulated islets

Immuno-isolation, hiding the islet grafts from recipients’ immune system, has become an effective strategy to protect pig islets from immune rejection (86). Till present, there are two types of immune-isolation devices: microencapsulation and macroencapsulation. Microencapsulated islets are microcapsules containing single islet or few islets, while the macrocapsules contain a few islets. The sizes of encapsulated islet grafts should be chosen according to implant sites as well as islet viability and function. Although the microcapsules are difficult to implant and remove, the permeability of microcapsules are better than that of macrocapsules. The ideal capsules should protect inner pig grafts from attacks mediated by host’s immune cells and enable free exchange of nutrients, oxygen, and wastes. Thus, the function of encapsulated islet is closely linked with biocompatibility of materials. (87). In recent years, a variety of artificial materials, including modified polysulfone, protamine–heparin complex, cellulose, agarose, ethylene glycol, and alginate were used to form macrocapsules or microcapsules, as a result, islet graft survival time was significantly prolonged (88–92). Moreover, after subcutaneous transplantation of encapsulated pig islets (alginate based), a 6-month correction of hyperglycemia was observed in diabetic NHPs without immunosuppression (14, 93).

Although many novel encapsulated pig islets have been developed and shown possible results in reducing xenogeneic rejection and prolonging functional graft survival time, several problems still exist before large-scale clinical, such as infectious complications, low diffusion capacity, and pericapsular fibrotic overgrowth.

### Co-stimulatory blocking

The engagement of TCR with foreign antigen without co-stimulatory signal will render T cells unresponsive to the antigen (known as T-cell anergy), thereby suppressing antigen induced response. Our previous study showed that the survival rate of donor-derived (pig) cytotoxic T lymphocyte antigen 4-immunoglobulin (CTLA4Ig) gene-modified islet xenografts was significantly prolonged in diabetic rats. The possible mechanism was that the CTLA4Ig fusion protein blocked CD28/B7 co-stimulatory signaling of the primary pathway, which eventually induced differentiation bias of T helper cells (Th cells) (94). When the direct and indirect pathways of T-cell activation were selectively blocked by pig CTLA4Ig modified immature dendritic cells and murine CTLA4Ig protein, the survival time of pig-islet xenografts was significantly prolonged (>100 days) in diabetic mice (95). Anti-CD154 antibodies, known to be effective in blocking indirect pathway of allorecognition (96, 97), is also a critical component of effective immunosuppressive strategies in preventing cellular rejection in pig-to-NHPs islet xenotransplantation (16, 98). However, the clinical application of anti-CD154 antibodies is restricted due to its high risk of thromboembolic complications (99). Notably, these co-stimulatory blockades have not induced immune tolerance, in which they are included in long-term immunosuppressive protocols. Similar to systemic immunosuppression, infection and morbidity are also detected in the recipients treated by co-stimulatory blocking. More specific co-stimulatory blockade should be conducted to improve the safety profile of tolerance induction.

### Gene-modified pig in islet xenotransplantation

Genetically modified pigs offer a number of potential advantages in minimizing the risk of thrombosis, reducing rapid loss of transplanted islets, decreasing the number of required islets, mitigating side effects of conventional/systemic immunosuppression, and improving islets activity and survival (6, 100, 101). Transgenic expression of human heme oxygenase-1 (HO-1) can effectively protect pig xenografts from ischemia/reperfusion injury and acute rejecting mediated by inflammatory cytokines (102) Humoral rejection can be overcome in pig-to-NHPs islet xenotransplant by crossbreeding of α1,3-galactosyltransferase gene-knockout (GT-KO) pigs with transgenic pigs expressing human complement regulators including CD46, CD59, and human decay-accelerating factor (hDAF, CD55) (103–105). Additional pig genetic engineering, knockout of TF, and overexpressing of human antithrombotic genes (CD39/thrombomodulin), will certainly prevent the occurrence of IBMIR and coagulation dysfunction (71). Pig islet transgenic for a high-affinity variant of CTLA4Ig also displays the potential to normalize glucose homeostasis and completely prevents cellular rejection in humanized mouse model (106). Recently, the development of RNA interference technology targeting porcine endogenous retroviruses (PERV) has substantially solved the possible problem of retrovirus contamination (107, 108). With the development and modification of genetic engineering, transgenic pigs will eventually drive islet xenotransplantation into clinical application.

## Other Factors Influencing Islet Survival

### Implant site

Successful pig-islet xenotransplantation is also closely related with appropriate selection of implantation site. The ideal transplant site should take into account: (1) surgical operation is simple and safe, (2) the ability to maintain a stable glucose metabolism, and (3) immune protection (109). Besides intrahepatic transplantation, renal subcapsular, subcutaneous, as well as omentum are commonly used sites in both experimental and preclinical islet xenotransplantation (110–112). Renal subcapsular and omentum represent the interesting alternatives due to advantages of relatively convenient and invasive process, sufficient blood and oxygen supply (omentum), portal venous drainage (omentum), and anatomical immune privilege.

### Islet graft revascularization

Regeneration of optimal microvascular supply is a vital prerequisite for islet transplantation (113). However, isolated pig islets are avascular and revascularization is generated 14 days after transplantation (114), therefore, promoting revascularization process and protecting newly formed microvasculature from rejection-mediated damage will immensely contribute to the improvement of islet function and survival. A recent study showed that, when islets were coated with mesenchymal stem cells (MSCs) and endothelial cells (ECs), the EC proliferation, sprout formation, migration of ECs into the islets as well as subsequent vascularization were significantly enhanced by MSCs (115). Similar findings were also demonstrated in syngeneic islet transplantation (116). Considering the powerful pro-angiogenic and immunomodulatory properties of MSCs, for pig-islet xenotransplantation, pretreatment of islet xenografts with recipient-derived MSCs will be helpful to accelerate islet revascularization and improve islet engraftment.

In addition, embryonic pig pancreatic tissue may also be another good choice. Embryonic pancreatic implants predominantly induce host-type vasculature to support growth and survival in diabetic rodents or monkeys (112, 117), thereby evading hyperacute or acute rejection.

## Clinical Study

The systematic clinical application of pig islets was first performed by Groth group (118). Between 1990 and 1993, 10 TIDM patients with kidney allografts were transplanted pig ICCs either intraportally or under the capsule of renal graft. After transplantation, pig C-peptide could be detected in the urine for 200–400 days in four patients. The data suggest that pig islets can survive in the humans, providing a good basis for clinical use of xenogeneic islet.

The long-term pig-islet viability and function was reported by Elliott et al. (15). The blood glucose level of T1DM patient was significantly reduced when the alginate-encapsulated NPIs was implanted intraperitoneally. After 10 years of follow-up, biopsy showed that there were still a large number of functional islets throughout peritoneal tissue. This single case study indicates that pig islets may have a positive long-term safety and therapeutic effect in the treatment of human T1DM, suggesting the necessity to conduct more large-scale clinical studies.

Living Cell Technology Co., Ltd. (LCT) developed a commercial encapsulated pig-islet product (Diabecell), which was tested in phase I/IIa clinical study in Moscow since 2007 (119, 120). A total of seven T1DM patients received Diabecell intra-abdominally at a dosage of 5,000–10,000 IEQ/kg, no significant adverse reactions were found post-transplantation. After 2-year follow-up, five patient’s blood glucose levels decreased to a normal range (5.8–8.2 mmol/L), two patients were independent with insulin administration. Additional I/IIa trials are being conducted in New Zealand.

## Safety of Porcine Islet Xenotransplantation

Interspecies transmission of PERV is still a potential risk factor in clinical pig-islet xenotransplantation. When human HK-293 cells were co-cultured with pig cells *in vitro*, PERV could infect human cells (121). In addition, the possibility of cross-species transmission of PERV was also confirmed in pig-to-SCID mice islet xenotransplantation (122). In contrast, no evidence of PERV activation was found in TIDM patients after long-term follow-up (123). However, PERV remains a potential threat requiring long-term follow-up in human clinical trials. Stringent PERV screening should be conducted in clinical islet xenotransplantation. With the emergence of PERV gene-knockout pigs, this bio-safety risk will be eliminated completely.

Besides PERV infections, other pathogens including herpesvirus, pig cytomegalovirus, lymphotropic herpesvirus, as well as bacterial pathogens also pose safety problems in pig-islet xenotransplantation, highlighting the importance of selecting of SPF pigs and prescreening of donor pigs.

## Conclusion

Building on the remarkable progress in the experimental/clinical studies, it appears that pig islets have grateful potentiality to reverse diabetes in NHPs and humans. With development of suitable sources of genetically modified pigs and modification of isolation technology, together with improvement of specific immunosuppressive methods, a tangible therapy will benefit the patients with diabetes in the very near future. However, questions remain and detailed problems need to be adequately addressed.

## Conflict of Interest Statement

The authors declare that the research was conducted in the absence of any commercial or financial relationships that could be construed as a potential conflict of interest.

## Abbreviations

α1,3GT, α1,3-galactosyltransferase; α-Gal, Galα1–3Galβ1–4GlcNAc-R; APCs, antigen presenting cells; APIs, adult pig islets; CMS, Chicago Medical School; CTLA4Ig, cytotoxic T lymphocyte antigen 4-immunoglobulin; ECs, endothelial cells; GMP, good manufacturing practices; GT-KO, α1,3-galactosyltransferase gene-knockout; HAR, hyperacute rejection; H–D antigen, Hanganutziu–Deicher antigen; IBMIR, instant blood-mediated inflammatory reaction; ICCs, islet-like cell clusters; IE/mm^2^, islet equivalents per mm^2^; IEQ, islet equivalents; MSCs, mesenchymal stem cells; NK cell, natural killer cell; NHPs, non-human primates; NPIs, neonatal pig islets; PERV, porcine endogenous retroviruses; SPF, specific pathogen-free; T1DM, type 1 diabetes mellitus; TCR, T-cell receptor; WIT, warm ischemia time.
